# Supplementary material for: Prevalence and causes of blindness and vision impairment in Western Uganda: Findings from a rapid assessment of avoidable blindness (RAAB) survey
Source: PLoS One. 2025 Oct 13;20(10):e0334509. doi: 10.1371/journal.pone.0334509 (PMC12517511; doi:10.1371/journal.pone.0334509)
Supplement: S1 Fig — (DOCX) [file pone.0334509.s001.docx]

**Supplemental Materials**

**Figure S1**. Comparison of survey population and census population by age and sex.


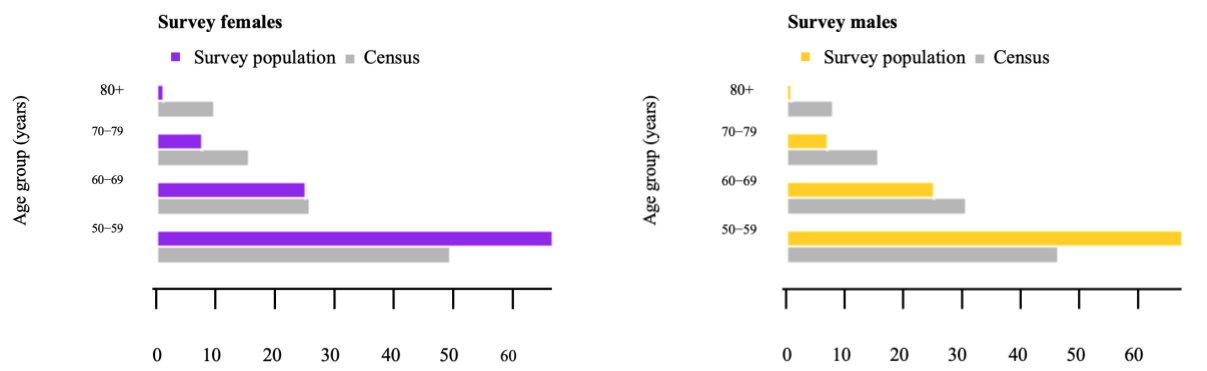


| **A.** |  |  | **B.** |  |  |  |  |  |  |  |
| --- | --- | --- | --- | --- | --- | --- | --- | --- | --- | --- |
| A represents survey females, and B represents survey males. | | | |  |  |  |  |  |  |  |

2
