# Supplementary material for: Prevalence and causes of blindness and vision impairment in Western Uganda: Findings from a rapid assessment of avoidable blindness (RAAB) survey
Source: PLoS One. 2025 Oct 13;20(10):e0334509. doi: 10.1371/journal.pone.0334509 (PMC12517511; doi:10.1371/journal.pone.0334509)
Supplement: S1 Table — (DOCX) [file pone.0334509.s002.docx]

**Supplemental Materials**

| **Table S1**. Principal cause of blindness and vision impairment by intervention category | | | | | | | | | | | |  |  |
| --- | --- | --- | --- | --- | --- | --- | --- | --- | --- | --- | --- | --- | --- |
|  | **Category** |  | **Blind, No** | |  | **Severe, No** | |  | **Moderate,** | |  | **Mild, No** |  |
|  |  |  |  | **(%)** |  |  | **(%)** |  | **No (%)** | |  | **(%)** |  |
|  | A. Treatable (1, 2, 3) | | 42 | (50.6) |  | 63 | (70.0) |  | 277 | (68.6) |  | 243 (86.8) |  |
|  | B. Preventable (PHC/PEC services) | | 5 | (6.0) |  | 1 | (1.1) |  | 4 (0.9) | |  | 1 (0.4) |  |
| (5, 6, 7, 8) | |  |  |  |  |  |  |  |  |  |  |  |  |
|  |  |  |  |  |  |  |  |  |  |  |  |  |  |
|  | C. Preventable (Ophthalmic | | 7 | (8.4) |  | 5 | (5.5) |  | 16 | (3.9) |  | 1 (0.4) |  |
|  | services) (4, 9, 10) | |  |  |  |  |  |  |  |  |  |  |  |
|  |  |  |  |  |  |  |  |  |  |  |  |  |  |
|  | D. Avoidable (A + B + C) | | 54 | (65.0) |  | 69 | (76.6) |  | 297 | (73.4) |  | 245 (87.6) |  |
|  | E. Posterior segment disease (8, | | 30 | (36.2) |  | 25 | (27.8) |  | 115 | (28.4) |  | 34 (12.1) |  |
| 9, 10, 11, 12, 13) | |  |  |  |  |  |  |  |  |  |  |  |  |
|  |  |  |  |  |  |  |  |  |  |  |  |  |  |

PHC: Primary Health Care; PEC: Primary Eye Care

**Treatable**: Uncorrected refractive error, uncorrected aphakia, and untreated cataract

**Preventable (PHC/PEC services)**: Trachomatous corneal opacity, other corneal opacities, phthisis, onchocerciasis

**Preventable (Ophthalmic services)**: Cataract surgical complications, glaucoma, diabetic retinopathy

**Posterior segment disease**: Onchocerciasis, glaucoma, diabetic retinopathy, age-related macular degeneration, other posterior segment diseases, myopic degeneration

2
