# Supplementary material for: Prevalence and causes of blindness and vision impairment in Western Uganda: Findings from a rapid assessment of avoidable blindness (RAAB) survey
Source: PLoS One. 2025 Oct 13;20(10):e0334509. doi: 10.1371/journal.pone.0334509 (PMC12517511; doi:10.1371/journal.pone.0334509)
Supplement: S2 Table — (DOCX) [file pone.0334509.s003.docx]

**Supplemental Materials**

**Table S2**. Post-operative visual outcomes after cataract surgery

|  |  | **Female, No (%)** | **Male, No (%)** | **Total, No (%)** |
| --- | --- | --- | --- | --- |
| **Presenting visual acuity (PVA)** | |  |  |  |
| Good (≥ 6/12) |  | 16 (23.5) | 5 (12.5) | 21 (19.4) |
| Borderline (<6/12 to 6/60) |  | 27 (39.7) | 20 (50.0) | 47 (43.5) |
| Poor (<6/60) |  | 25 (36.8) | 15 (37.5) | 40 (37.0) |
| **Pinhole visual acuity (PinVA)** | |  |  |  |
| Good (6/12) |  | 26 (38.2) | 15 (37.5) | 41 (38.0) |
| Borderline (<6/12 ≥ 6/60) |  | 23 (33.8) | 13 (32.5) | 36 (33.3) |
| Poor (<6/60) |  | 19 (27.9) | 12 (30.0) | 31 (28.7) |

*Blindness=PVA <3/60 in the better seeing eye. Severe VI=PVA ≥ 3/60 but <6/60 in the better seeing eye. Moderate VI=PVA ≥ 6/60 but <6/18 in the better seeing eye. Mild VI=PVA ≥ 6/18 but <6/12 in the better seeing 2
