# Supplementary material for: Prevalence and causes of blindness and vision impairment in Western Uganda: Findings from a rapid assessment of avoidable blindness (RAAB) survey
Source: PLoS One. 2025 Oct 13;20(10):e0334509. doi: 10.1371/journal.pone.0334509 (PMC12517511; doi:10.1371/journal.pone.0334509)
Supplement: S3 Table — (DOCX) [file pone.0334509.s004.docx]

**Supplemental Materials**

**Table S3**. Age- and sex- adjusted distance refractive error coverage and effective refractive error coverage

|  | **Female % (95% CI)** | **Male % (95% CI)** | **Total % (95% CI)** |
| --- | --- | --- | --- |
| eREC | 0.0 (0.0 – 0.0) | 1.3 (0.0 – 3.4) | 0.6 (0.0 – 1.4) |
| REC | 0.0 (0.0 – 0.0) | 2.2 (0.0 – 4.7) | 1.0 (0.0 – 2.0) |

eREC: effective refractive error coverage; REC: refractive error coverage

2
