# Supplementary material for: Prevalence and causes of blindness and vision impairment in Western Uganda: Findings from a rapid assessment of avoidable blindness (RAAB) survey
Source: PLoS One. 2025 Oct 13;20(10):e0334509. doi: 10.1371/journal.pone.0334509 (PMC12517511; doi:10.1371/journal.pone.0334509)
Supplement: Attachment — Submitted filename: PONE-D-25-28213_Reviewer comments to authors.doc [file pone.0334509.s005.doc]

| **PONE-D-25-28213** | |
| --- | --- |
| **Prevalence and causes of blindness and vision impairment in Western Uganda: Findings from a rapid assessment of avoidable blindness (RAAB) survey** | |
| **General observation** | It is great to see evidence being generated from the African region, where data remain limited for planning. Truly appreciate the efforts of the research team.   Overall, the article is presented well. Areas for improvement include: providing clarity on the eye examination process; avoiding repetition of definitions to enhance readability; and strengthening the Discussion section. |
| **Abstract** |  |
| Methods | Participants, sampling, and recruitment details should be included. |
|  | RAAB version, whether 6 or 7, may be mentioned. |
| Results | Add total participants and sex details. |
|  | CSC and eCSC should be reported at 6/12 threshold, as this is readily available in the RAAB7 version. Additional thresholds may be reported in the main text as appropriate. Reporting at 6/12 threshold will further support the conclusion in the abstract. |
|  | eREC should be defined either in the methods or results section. |
| Keywords | eCSC, eREC may be added. |
| **Introduction** |  |
| Line no: 83 | a shortage of trained eye care providers':-  The word ‘provider’ word can be replaced with 'professionals' if you refer to eye care workforce. If you are referring to an eye hospital, then 'trained' word might be removed. |
| Line no: 86 -88 | The leading cause of blindness globally is cataract, with an estimated 17.01 million people, followed by uncorrected refractive error with an estimated 3.70 million people:-  Please recheck the burden count in the article you cited.  Also mention the age group. |
| **Materials and methods** |  |
| Line no: 150 | Eye examination would be an appropriate word instead of the subheading 'study procedure'. |
| Line no: 150-154 | Uncorrected visual acuity (UCVA) was measured with both eyes open using a tumbling E-chart.' As per RAAB protocol, visual acuity is measured for one eye first (while covering the other eye), followed by next eye. Kindly clarify.   'In both groups, when VA was <6/12, the 153 visual acuity with pinhole testing (PinVA) was performed to assess for best-corrected visual acuity (BCVA).' For pinhole criteria, specify whether it is based on UCVA/PVA |
| Line no: 155-156 | Check for grammatical accuracy. |
| Line no: 163 | In RAAB7 version, 'name' is not collected for all individuals, but only for the unavailable persons. However, these names were saved only in local tablets during data collection, not saved in RAAB database. Hence this can be removed from demography.   'optional GPS coordinates':- You may mention this only if you used this variable. Otherwise, not necessary to mention. |
| Line no: 175 | and London School of Hygiene & Tropical Medicine Observational Ethics Committee'  Individual RAAB survey do not require ethical approval from LSHTM; the research team only makes an agreement for use of RAAB7 software. Therefore, this statement should be not included under ethics section. |
| **Results** |  |
| Line no: 182-191 | Table 1 is cited in line no 184. It is assumed that the subsequent sentences refer to the same. So there is no need to repeat citing table no. for each sentence. |
| Line no: 195 -200 | Definition of blindness and VI is provided in the Methods section and in the accompanying table. Repetition may be avoided where possible. |
| Line no: 222-226 | cataract surgical coverage is highlighted at 6/60 threshold, rather than the meaningful 6/12 threshold, which is essential and recommended by WHO as the benchmark that countries should aim to achieve. Moreover, you have already discussed the 2030 WHO targets in the Introduction. |
| Line no: 228 | Quality gap can be included in Table 4 |
| Line no: 235 | Total 'n' counts can be given in Table 5 Total counts may be provided in all tables in the last row, where possible. |
| Line no: 245-247 | Total refractive error prevalence can be mentioned, though a breakdown of VI was given. |
| **Discussion** | Nearly half of the Discussion section is repetition of results. There is scope for strengthening this section by focusing on interpretation, implications, recommendations to eye care providers, government, NGOs. |
| Line no: 266-268 | The comparison statement seems to be incorrect. Please be careful while quoting other studies. The following is the prevalence % from the article you cited.....'Prevalence of visual impairment was found to have decreased from 6.0% (95%CI 5.2-6.9%) in 2015 to 4.9% (95%CI 4.0-5.9%]) in 2023' |
| Line no: 270-272 | Unlike in 2013, 271 where a significantly higher proportion of blindness from untreated cataract was observed in females (62.9% vs. 32.1%), our study found similar rates between sexes (47.9% vs. 51.4%).'  You can say that this difference was due to various initiatives of cataract programs....list programs. |
| Line no: 273 | Mention visual acuity threshold for 2013 RAAB |
| Line no: 297-299 | Compared to the 2013 RAAB, where cataract surgeries were predominantly performed in government hospitals [18], our study found a nearly equal distribution of procedures across government, non-governmental organization (NGO), and private hospitals.'  In Results section, there is no statement regarding the place of surgery, therefore, it should not discussed in the Discussion section. |
