# Supplementary material for: Prevalence and causes of blindness and vision impairment in Western Uganda: Findings from a rapid assessment of avoidable blindness (RAAB) survey
Source: PLoS One. 2025 Oct 13;20(10):e0334509. doi: 10.1371/journal.pone.0334509 (PMC12517511; doi:10.1371/journal.pone.0334509)
Supplement: Attachment — Submitted filename: Response to Reviewers.docx [file pone.0334509.s007.docx]

**Journal Requirements**:

1. Please ensure that your manuscript meets PLOS ONE's style requirements, including those for file naming.

**Response**: Our manuscript meets the PLOS One style requirements.

2. Thank you for stating the following financial disclosure: “This research was funded by Christian Blind Mission (CBM), Uganda Office.” Please state what role the funders took in the study.  If the funders had no role, please state: "The funders had no role in study design, data collection and analysis, decision to publish, or preparation of the manuscript." If this statement is not correct you must amend it as needed. Please include this amended Role of Funder statement in your cover letter; we will change the online submission form on your behalf.

**Response**: This research was funded by Christian Blind Mission (CBM), Uganda Office. The funders had no role in study design, data collection and analysis, decision to publish, or preparation of the manuscript.

3. Please confirm at this time whether or not your submission contains all raw data required to replicate the results of your study. Authors must share the “minimal data set” for their submission. PLOS defines the minimal data set to consist of the data required to replicate all study findings reported in the article, as well as related metadata and methods (<https://journals.plos.org/plosone/s/data-availability#loc-minimal-data-set-definition>).

**Response**: All the required data to replicate the results of the study have been provided in the manuscript.

4. If the reviewer comments include a recommendation to cite specific previously published works, please review and evaluate these publications to determine whether they are relevant and should be cited. There is no requirement to cite these works unless the editor has indicated otherwise. 
**Response**: Done.

5. Please review your reference list to ensure that it is complete and correct. If you have cited papers that have been retracted, please include the rationale for doing so in the manuscript text, or remove these references and replace them with relevant current references. Any changes to the reference list should be mentioned in the rebuttal letter that accompanies your revised manuscript. If you need to cite a retracted article, indicate the article’s retracted status in the References list and also include a citation and full reference for the retraction notice.
**Response**: Done.

**Additional Editor Comments (if provided)**:

Dear Authors,

Congratulations on a very well written paper. It is especially important to generate the evidence and base line data in a region where there is a paucity of recent population-based studies conducted. It will help the country to determine the steps needed to fulfill the targets set out by the WHA resolutions.

**Response**: We thank the Editor for the encouraging feedback and are pleased the study is recognized as a valuable contribution to addressing evidence gaps and supporting progress toward WHA targets.

**Reviewers' comments**:

**Comments to the Author**

*1. Is the manuscript technically sound, and do the data support the conclusions?*

Reviewer #1: Yes

Reviewer #2: Yes

**Response**: Thank you.

*2. Has the statistical analysis been performed appropriately and rigorously?*

Reviewer #1: Yes

Reviewer #2: Yes

**Response**: Thank you.

*3. Have the authors made all data underlying the findings in their manuscript fully available?*

Reviewer #1: Yes

Reviewer #2: Yes

**Response**: Thank you.

*4. Is the manuscript presented in an intelligible fashion and written in standard English?*

Reviewer #1: Yes

Reviewer #2: Yes

**Response**: Thank you.

*5. Review Comments to the Author*

**Reviewer #1**: I think this is a very well set up study. I really appreciate the hard field work the ophthalmologists have done in their house-to-house visits to collect this data. They adhered well to the RAAB study set up. I would consider a small rewrite of the discussion. It is stating arguments on the reasons for an ineffective cataract surgical coverage and is stating that there were no reviews of surgeries done within 3 days and no proper biometry which might have led to poor refractive outcomes. These are however suggestions and these data were not collected in questionnaires on the patients who underwent cataract surgery. Nor was refraction done on those patients. So, I suggest keeping those arguments out of the discussion. Or rewrite them as possible reasons as shown in other studies, with references.

**Response**: We thank the Reviewer for the positive feedback and for recognizing the extensive field work undertaken. We also appreciate the insightful suggestion regarding the discussion. We agree that our current wording may imply that these factors were directly observed in our cohort. In line with the Reviewer’s advice, we have revised the discussion to present these as possible explanations reported in other studies, with appropriate references:

Line 294-306: “Several strategies, identified in other settings, could enhance cataract surgery outcomes in Western Uganda, though their effectiveness in this population remains uncertain.

First, structured post-surgical monitoring is strongly associated with better results [22]. In settings with poor follow-up, early VA assessment within three days of surgery, and unprompted assessments of patients returning after 6 weeks are considered valid measures of operative quality [23]. This monitoring should be conducted at the individual surgeon or center level, not for direct comparisons, but to identify areas for improvement [24], including better selection of surgical candidates. The extent of follow-up among participants in our study remains unclear, as this was not assessed.

Second, investments in optical biometry and enhanced surgical training could optimize outcomes and minimize deviations from target refraction [25, 26]. Finally, human resource constraints remain a significant challenge, with the most recent data revealing one ophthalmologist per 1.2 million [27], and two ophthalmology residency training programs in Uganda [5]. Beyond increased ophthalmologists, optometrist, and allied ophthalmic personnel, there remains a need for enhanced surgical training [28].”

**Reviewer #2**:  It is great to see evidence being generated from the African region, where data remain limited for planning. Truly appreciate the efforts of the research team. Overall, the article is presented well. Areas for improvement include providing clarity on the eye examination process; avoiding repetition of definitions to enhance readability; and strengthening the Discussion section.
**Response**: We thank the Reviewer for their kind comments. We have clarified the eye examination process, removed repeated definitions, and strengthened the Discussion section as suggested. These changes have been detailed in response to your later comments, to avoid repetition.

**ABSTRACT**:

**Methods**: Participants, sampling, recruitment details should be included. RAAB version, whether 6 or 7, may be mentioned.
**Response**: We thank the Reviewer for this suggestion. We have now added details on participants, sampling, and recruitment briefly (given word-count limitations) in the abstract, and specified that RAAB7 was used:

Line 48 – 53: “Methods: A population-based cross-sectional survey was conducted in Western Uganda (July-August 2023) using RAAB7. Adults aged ≥50 years who had resided in the study districts for at least six months in the past year were eligible. Participants were identified through door-to-door household visits using a two-stage cluster sampling approach.”

**Results**: Add total participants and sex details. CSC and eCSC should be reported at 6/12 threshold, as this is readily available in the RAAB7 version. Additional thresholds may be reported in the main text as appropriate. Reporting at 6/12 threshold will further support the conclusion in the abstract. eREC should be defined either in methods or results section.

**Response**: We have included the total number of participants with sex distribution. CSC and eCSC are now reported at the 6/12 threshold in the Abstract, with additional thresholds presented in the main text. eREC has also been defined in the Abstract Methods section.

Line 51 – 53: “Primary outcomes include prevalence of blindness and VI and its causes. Secondary outcomes include cataract surgical coverage (CSC), effective CSC (eCSC), refractive error coverage (REC), and effective REC (eREC).”

Line 55 – 63: “A total of 3,125 participants were examined (54.1% female). The adjusted prevalence of blindness (presenting visual acuity (PVA) <3/60) was 0.9% (95% CI: 0.5–1.3%). Severe, moderate, and mild VI were found in 0.9% (95% CI: 0.4–1.3%), 4.5% (95% CI: 3.3–5.8%), and 3.8% (95% CI: 3.0–4.6%), respectively. Untreated cataract was the leading cause of bilateral blindness (49.4%). The CSC and eCSC at the <6/12 threshold were 19.7% and 7.3%, respectively.. Only 19.4% of 108 operated eyes achieved good outcomes (PVA ≥6/12). The main barriers to cataract surgery included lack of awareness (32.8%), cost (23.9%), and perceived lack of need (20.9%). The adjusted prevalence of uncorrected refractive error as a cause of moderate VI was 1.6% (95% CI: 1.1–2.0%), and mild VI was 2.8% (95% CI: 2.2–3.5%). REC was 1.0%, while eREC was 0.6% (95% CI: 0.0–1.4%).”

**Keywords**: eCSC, eREC may be added.
**Response**: Thank you for your suggestion. We have added “eCSC” and “eREC” as keywords.

Line 70 – 71: “Vision screening; vision disorders; blindness; prevalence; health equity; cataract; refractive errors; RAAB; Uganda; CSC; eCSC; REC; eREC”

**INTRODUCTION**:

**Line no**: 83 a shortage of trained eye care providers':- The provider word can be replaced with 'professionals' if you refer to eye care workforce. If you are referring to eye hospital, then 'trained' word might be removed.
**Response**: We thank the reviewer for this observation. We have revised the wording to better reflect the intended reference to the eye care workforce:

Line 82 – 85: “The reasons for this are multifactorial [3], including a shortage of trained eye care professionals [4], a limited number of ophthalmology training programs [5], and existing barriers preventing individuals from presenting for treatment [6].”

**Line no**: 86 -88 The leading cause of blindness globally is cataract, with an estimated 17.01 million people, followed by uncorrected refractive error with an estimated 3.70 million people':- Please recheck the burden count in the article you cited. Also mention the age group.
**Response**: We have rechecked the cited source and corrected the global burden figures accordingly. We have also specified the age group (≥50 years) in the revised sentence:

Line 86 – 88: “Cataract is the leading cause of blindness worldwide among adults aged ≥50 years, affecting an estimated 15.2 million people, followed by glaucoma (3.6 million) and uncorrected refractive error (2.3 million) [8].”

**MATERIALS AND METHODS**:

**Line no**: 150 Eye examination would be an appropriate word instead of the subheading 'study procedure'.
**Response**: We have revised the subheading “Study procedure” to “Eye examination” (Line 150) as suggested.

**Line no**: 150-154 ‘Uncorrected visual acuity (UCVA) was measured with both eyes open using a tumbling E-chart.' As per RAAB protocol, visual acuity is measured for one eye first (while covering the other eye), followed by next eye. Kindly clarify. 'In both groups, when VA was <6/12, the visual acuity with pinhole testing (PinVA) was performed to assess for best-corrected visual acuity (BCVA).' For pinhole criteria, specify whether it is based on UCVA/PVA

**Response**: We have clarified that, following RAAB protocol, visual acuity was measured in each eye separately, covering the other eye during testing. Furthermore, we have specified when pinhole visual acuity (PinVA) was performed:

Line 150 – 156: “*Eye examination*. Uncorrected visual acuity (UCVA) was measured in each individual eye, using a tumbling E-chart. Individuals who habitually wear spectacle correction for distance vision had their corrected visual acuity (CVA) re-measured with spectacles on. In both groups, when VA (UCVA and/or CVA) was <6/12, the visual acuity with pinhole testing (PinVA) was performed to assess for best-corrected visual acuity (BCVA).

Presenting visual acuity (PVA) was defined as UCVA for participants without spectacles, and CVA for participants who habitual wear distance correction, as per RAAB7 protocol.”

**Line no**: 155-156 Check for grammatical accuracy.
**Response**: The sentence has been revised for grammatical accuracy:

Line 153 – 154: “Presenting visual acuity (PVA) was defined as UCVA for participants without spectacles, and CVA for participants who habitual wear distance correction, as per RAAB7 protocol.”

**Line no**: 163 "In RAAB7 version, 'name' is not collected for all individuals, but only for the unavailable persons. However, these names were saved only in local tablets during data collection, not saved in RAAB database. Hence this can be removed from demography. 'optional GPS coordinates':- You may mention this only if you used this variable. Otherwise, not necessary to mention. "
**Response**: Thank you for this suggestion. We have removed references to participant names and optional GPS coordinates, as these were not included in the RAAB database or used in analysis.

Line 162 – 163: “In addition to PVA, each participant underwent a structured ocular history and examination, including: (i) participant demographics (age, sex, spectacle use history)”

**Line no**: 175 "and London School of Hygiene & Tropical Medicine Observational Ethics Committee'; Individual RAAB survey do not require ethical approval from LSHTM; the research team only makes an agreement for use of RAAB7 software. Therefore, this statement should be not included under ethics section.
**Response**: We have removed the statement regarding LSHTM ethics approval, as suggested. The ethics approval statement now reads as follows:

Line 173 – 175: “Ethical approval was obtained from the Mulago Hospital Research and Ethics Committee (MHREC), and Uganda National Council for Science and Technology (UNCST).”

**RESULTS**:
**Line no**: 182-191 Table 1 is cited in line no 184. It is assumed that the subsequent sentences refer to the same. So, there is no need to repeat citing table no. for each sentence.
**Response**: We have removed repeated citations of Table 1 within the same paragraph, as suggested, to improve readability:

Line 181 – 188: “A total of 4,895 eligible adults aged 50 years and older were identified for participation. Of these, 4,725 individuals were successfully examined, yielding a response rate of 96.5% (Table 1). The response rate was similar between males (96.3%) and females (96.7%). Among the non-responders, 1.9% were unavailable, 1.0% were incapable of participating, and 0.7% refused. The study sample comprised 2,572 (54.4%) females and 2,153 (45.6%) males. The age distribution was 47.9% aged 50–59 years, 27.9% aged 60–69 years, 15.4% aged 70–79 years, and 8.8% aged 80+ years. Compared to the census population, males in the younger age group (50–59 years) were slightly underrepresented, whereas females in the older age groups (≥70 years) were overrepresented (Supplemental Material, Figure S1).”

**Line no**: 195 -200 Definition of blindness and VI is provided in the Methods section and in the accompanying table. Repetition may be avoided where possible.
**Response**: Thank you for your suggestion. We have avoided repeating definitions of blindness and visual impairment, given they are already provided in the Methods section and tables.

**Line no**: 222-226 cataract surgical coverage is highlighted at 6/60 threshold, rather than the meaningful 6/12 threshold, which is essential and recommended by WHO as the benchmark that countries should aim to achieve. Moreover, you have already discussed the 2030 WHO targets in the Introduction.
**Response**: We have added cataract surgical coverage (CSC) at the 6/12 threshold as suggested, in line with WHO recommendations and our discussion of 2030 WHO targets:

Line 218 – 221: “*Cataract surgical coverage*. The age- and sex- adjusted cataract surgical coverage (CSC) at the PVA <6/12 threshold was 14.9% overall (Table 4). However, effective cataract surgical coverage (eCSC), which represents the number of people who have been operated on and had good surgical outcomes (PVA ≥6/12), was 5.1%, highlighting a 65.8% relative quality gap.”

**Line no**: 228 Quality gap can be included in Table 4
**Response**: We have included the quality gap data in Table 4 (Line 221), as suggested.

**Line no**: 235 Total 'n' counts can be given in Table 5. Total counts may be provided in all tables in the last row, where possible.
**Response**: Total counts have been added to the final row of tables, where possible, including Table 5 (Line 230), Table 3 (Line 212), and Table 1 (Line 190).

**Line no**: 245-247 Total refractive error prevalence can be mentioned, though breakdown of VI was given.
**Response**: Thank you for your suggestion. We have added the extrapolated magnitude of refractive error in Table 6 (Line 249), in addition to including this in the manuscript body, as suggested:

Line 240 – 244: “The age- and sex-adjusted prevalence and extrapolated magnitude of uncorrected refractive error as a cause of moderate VI was 1.6% (95% CI: 1.1–2.0%, N=2,160) and mild VI was 2.8% (95% CI: 2.2–3.5%, N=3,903) (Table 6). Refractive error coverage (REC) was 1.0% overall, while effective refractive error coverage (eREC) was only 0.6% (95% CI: 0.0–1.4%), reflecting a significant gap in refractive services (Supplemental Material, Table S3).”

**DISCUSSION**:
**Comment**: Nearly half of the Discussion section is repetition of results. There is scope for strengthening this section by focusing on interpretation, implications, recommendations to eye care providers, government, NGOs.
**Response**: We have substantially revised the Discussion section to reduce repetition of results and enhance interpretation, implications, and actionable recommendations. The revised text contextualizes the prevalence of blindness and severe vision impairment relative to other regions, highlights gender equity in cataract surgery uptake, and emphasizes ongoing gaps in cataract surgical quality and refractive services.

**A. Discussion of interventions to improve gender equity in cataract surgery access and uptake**

Line 269 – 281: “This may reflect improvements in equity of access, resulting from strategies designed to enhance women’s access to cataract surgery, including community engagement, service delivery adaptations, and gender-responsive planning [20]. Community approaches included working with women’s groups, community health workers, and household decision-makers to raise awareness and mobilize women, while outreach and surgical services were adapted through women-only camps, priority management for pregnant or breastfeeding women, gender-segregated waiting areas, same-day surgery, and transport or financial support to reduce indirect barriers. Programmes also used sex-disaggregated baseline data and gender-specific targets to inform planning, complemented by capacity building for health workers and ongoing monitoring of gender-disaggregated outcomes. Systematic case finding, school-based health education, and documentation of best practices further supported equitable service delivery, addressing both practical and societal barriers to surgical uptake among women [20]. Ongoing efforts are needed to ensure all populations, particularly women and older adults, can access high-quality cataract services.”

**B. Discussion of prioritization of cataract surgical quality over volume, and potential strategies**

Line 290 – 305: “Despite these considerations, the cataract quality gap in this study remains substantial at 64.6%. Being well over 25% threshold, this emphasizes that improving surgical quality should take precedence over simply increasing surgical volume [21], as enhanced quality directly impacts functional outcomes. Several strategies, identified in other settings, could enhance cataract surgery outcomes in Western Uganda, though their effectiveness in this population remains uncertain.

First, structured post-surgical monitoring is strongly associated with better results [22]. In settings with poor follow-up, early VA assessment within three days of surgery, and unprompted assessments of patients returning after 6 weeks are considered valid measures of operative quality [23]. This monitoring should be conducted at the individual surgeon or center level, not for direct comparisons, but to identify areas for improvement [24], including better selection of surgical candidates. The extent of follow-up among participants in our study remains unclear, as this was not assessed.

Second, investments in optical biometry and enhanced surgical training could optimize outcomes and minimize deviations from target refraction [25, 26]. Finally, human resource constraints remain a significant challenge, with the most recent data revealing one ophthalmologist per 1.2 million [27], and two ophthalmology residency training programs in Uganda [5]. Beyond increased ophthalmologists, optometrist, and allied ophthalmic personnel, there remains a need for enhanced surgical training [28].”

**C. Discussion of factors associated with poor surgical uptake, interventions that improve uptake, and need for further research**

Line 311 – 326: “The main barriers to cataract surgery in our study were lack of awareness that treatment was available (32.8%), cost (23.9%), and fear of surgery (14.9%). These findings are consistent with previous RAABs in Uganda [14, 18], highlighting the persistent need for public health education to promote cataract surgery uptake [30]. A study at Mulago National Referral Hospital in Kampala, Uganda, found that financial constraints, the perception that surgery was unnecessary, and the ability to see with one eye were the primary reasons for delayed cataract surgery among operable patients [31]. Several factors have been associated with higher cataract surgery uptake [6], including male sex [32, 33], relatively younger age (50–60 years vs. >70 years) [33], and supportive family attitudes toward surgery [34], although other studies have not found the same significant associations [35-38]. Interventions shown to improve uptake include eliminating direct surgical costs [32]; however, even when surgery is free, uptake may remain lower than expected [33]. This may be due to indirect cost such as transportation, and lost income from time off work [39, 40]. Regular outreach programs have also been reported to increase uptake [37, 41], though it is unclear whether this effect reflects reduced travel barriers or greater trust in familiar service delivery models [6]. Further research is needed to move beyond self-reported barriers and evaluate interventions in real-world clinical settings to determine which strategies effectively improve cataract surgery uptake.”

**Line no**: 266-268 The comparison statement seems to be incorrect. Please be careful while quoting other studies. The following is the prevalence % from the article you cited.....'Prevalence of visual impairment was found to have decreased from 6.0% (95%CI 5.2-6.9%) in 2015 to 4.9% (95%CI 4.0-5.9%]) in 2023'
**Response**: Thank you for pointing this out. The prevalence figures from the cited study have been corrected:

Line 262 – 264: “In contrast, Karamoja, a region in Northern Uganda with one of the highest national poverty rates [19], reported a 2023 prevalence of blindness and severe VI of 4.9% and 3.1%, respectively [14], highlighting significant regional differences likely influenced by economic factors.”

<https://www.raab.world/survey/uganda-northern-karamoja-2023#results>

**Line no**: 270-272 “Unlike in 2013, when a significantly higher proportion of blindness from untreated cataract was reported in women (CSC <3/60: 70.9% in men vs. 37.4% in women) [18], our study found more comparable rates between sexes (CSC <3/60: 50.8% in men vs. 67.1% in women).” You can say that this difference was due to various initiatives of cataract programs....list programs.
**Response**: Thank you for your suggestion. We revised the text to include the impact of various cataract programs, as suggested:

Line 269 – 281: “This may reflect improvements in equity of access, resulting from strategies designed to enhance women’s access to cataract surgery, including community engagement, service delivery adaptations, and gender-responsive planning [20]. Community approaches included working with women’s groups, community health workers, and household decision-makers to raise awareness and mobilize women, while outreach and surgical services were adapted through women-only camps, priority management for pregnant or breastfeeding women, gender-segregated waiting areas, same-day surgery, and transport or financial support to reduce indirect barriers. Programmes also used sex-disaggregated baseline data and gender-specific targets to inform planning, complemented by capacity building for health workers and ongoing monitoring of gender-disaggregated outcomes. Systematic case finding, school-based health education, and documentation of best practices further supported equitable service delivery, addressing both practical and societal barriers to surgical uptake among women [20]. Ongoing efforts are needed to ensure all populations, particularly women and older adults, can access high-quality cataract services.”

**Line no**: 273 Mention visual acuity threshold for 2013 RAAB
**Response**: The visual acuity threshold for the 2013 RAAB has been specified (<6/60 threshold), as suggested.

**Line no**: 297-299 ‘Compared to the 2013 RAAB, where cataract surgeries were predominantly performed in government hospitals [18], our study found a nearly equal distribution of procedures across government, non-governmental organization (NGO), and private hospitals.' In Results section, there is no statement regarding the place of surgery, therefore, it should not discussed in the Discussion section.
**Response**: We agree that as the Results section does not report on place of surgery, the discussion regarding location of surgery is not appropriate, and has thus been removed.
